# Fixation for metaphyseal-diaphyseal junction noncomminuted fracture of the distal humerus in children: K-wire or ESIN, how to decide?

**DOI:** 10.3389/fped.2025.1640764

**Published:** 2025-08-06

**Authors:** Minglei Li, Tianjing Liu, Qiwei Li, Lianyong Li, Lijun Zhang, Liwei Shi, Enbo Wang

**Affiliations:** Department of Pediatric Orthopedics, Shengjing Hospital of China Medical University, Shenyang, China

**Keywords:** metaphyseal-diaphyseal junction fracture, closed reduction and percutaneous pinning, elastic stable intramedullary nail, external fixator, humerus, pediatric

## Abstract

**Background:**

The metaphyseal-diaphyseal junction (MDJ) fracture of the distal humerus has posed significant difficulty clinically, as the increased height of the distal fragmant makes it hard for Kirschner wires to reach the proximal fragment. Our previous study provided suggestions for the choice of fixation in metaphyseal-diaphyseal junction (MDJ) fracture of the distal humerus according to the location of the fracture line based on biomechanical analysis. This study went on to testify an advanced suggestions in clinical patients.

**Methods:**

Normal elbow x-rays were measured to get a normal reference value to define the location of the fracture. A ratio of c' (the diameter of humeral shaft at the most proximal point of the fracture line)/d (the diameter of humeral shaft at distal humerus) was used to define the location of the fracture and guide the selection of fixation. According to our previous research, the ratio of c′/d was used to define the location of the fracture. Eighty-nine patients with MDJ fractures were included. For patients with high MDJ fracture elastic stable intramedullary nails (ESIN) were selected and for those with low MDJ fractures Kirschner wires were used. The short-term outcome was assessed using the Flynn criteria.

**Results:**

The c/d ratio of 1.2 was finally used to define the high or low location of the fracture. All the 89 MDJ fractures healed uneventfully. 73 of them were fixed with lateral or crossed pinning and 84.9% of them were ranked as excellent. 16 cases were fixed with ESIN and 81.3% were excellent. There were no significant difference between the outcomes of the groups.

**Conclusions:**

ESINs were used for fractures in the higher part of the MDJ region, defined as c′/d < 1.2. Three lateral divergent or crossed pins were used for fractures in the lower part of the MDJ region with c′/d ≥ 1.2. This strategy, as recommended by our previous biomechanical research, has been demonstrated to be practical in clinical practice.

**Level of evidence:**

Level III retrospective cohort study.

## Introduction

The metaphyseal-diaphyseal junction (MDJ) fracture of the distal humerus, which used to be regarded as a particular type of humeral supracondylar fracture, has always been challenging to the surgeons (1). It accounts for about 3.3% of all humeral supracondylar fractures (2). Fayssoux classified the fracture line of MDJ fractures as transverse and lateral oblique (2). The third type, the comminuted type, was added by Sen et al. (3). For displaced MDJ fractures that require internal fixation, the increased lever arm makes the conventional fixation strategy with Kirschner wires difficult or even impossible. Other strategies for humeral supracondylar fractures, such as external fixators (EF) and elastic stable intramedullary nails (ESIN) have also been suggested, but there has been no management principle or even consensus on the ideal fixation strategy for MDJ fractures (4–6).

Our previous biomechanical study investigated the stability of external fixators, ESIN, lateral divergent and crossed pinning in MDJ fractures (7). ESIN yields the best stability in fixing fractures in the upper part of the MDJ region, while three crossed (one medial and two lateral) pinning can achieve the best stability for fractures in the lower part of the MDJ region (7). However, that study was done on composite bones that might not reflect clinical reality. Besides, the upper and lower regions of the MDJ might not be easily defined when the fracture is severely displaced.

In this study, we went on to test our findings in clinical cases. First, normal elbow radiographs were measured to get a reference value for the definition of fracture locations. Then the internal fixations for displaced MDJ fractures were selected according to a combination of the normal reference and the results of our previous studies. The patients were closely followed-up and their short-term outcomes were reported. We aimed at offering a quantified, practical rationale for fixation selection of humeral MDJ fractures.

## Materials and methods

**Figure 1:**
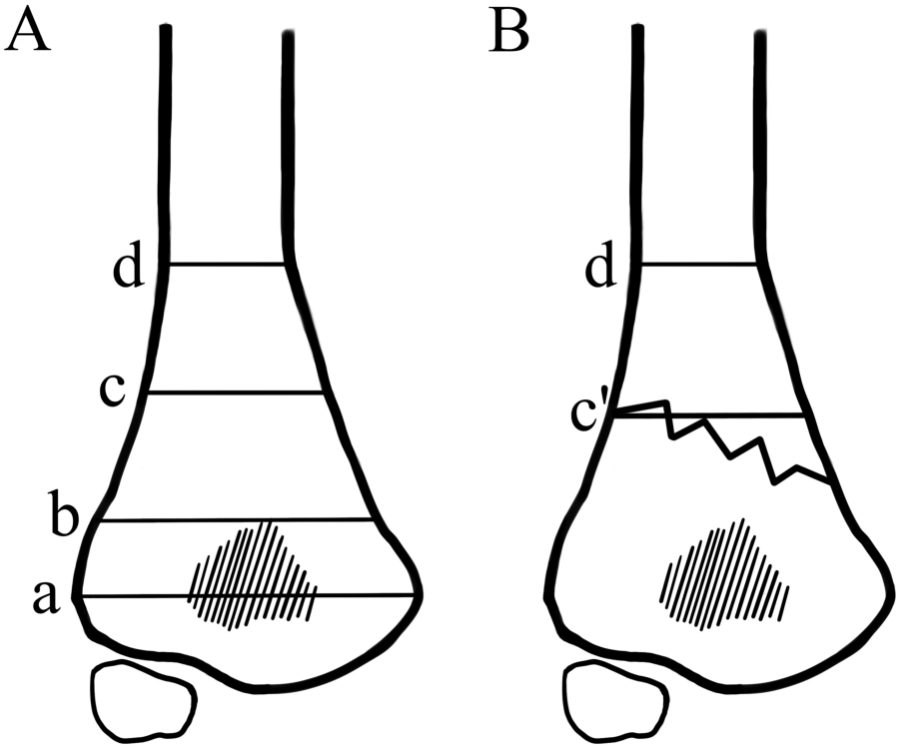
Illustrations of the a/d, b/d and c/d ratio. **(A)** Line a: the widest inter-condylar distance between the lateral and the medial condyles; Line b: the horizontal line at the top of the olecranon fossa; Line d: a horizontal line passing through the level where the humeral shaft starts to widen; Line c: a line that goes midway between b and d, which separating the MDJ region as the higher half and the lower half. The ratio of c/d was calculated by dividing the length of line c with that of line d. **(B)** Line c′ is a parallel line to line d that was drawn at the higher intersection point of the fracture line and the margin of the humeral shaft. The ratio of c′/d was calculated by dividing the length of line c′ with that of line d.

This study had been approved by the ethics committee of the XXX Hospital. Normal antero-posterior (AP) radiographs of the elbow in children aging 1–14 from 2016 to 2020 were collected to obtain normal reference values. Those radiographs were taken to exclude bone injury and had been proven to be normal by two radiologists and one pediatric orthopedician. Line a represented the widest inter-condylar distance between the lateral and the medial condyles (Figure 1). The MDJ region was defined as the space between line b (a horizontal line drawn at the top of the olecranon fossa) and line d (a horizontal line passing through the level where the humeral shaft starts to widen). A line (Line c) was drawn midway between b and d, which separating the MDJ region as the higher half and the lower half. The ratio of the a/d (the length of a divided by the length of d), b/d (the length of b divided by the length of d) and c/d (the length of c divided by the length of d) were calculated and summarized as a reference (Figure 1A).

We investigated pediatric patients that had been surgically treated for displaced MDJ fractures in our institute between January 2016 and December 2022. They had been treated with closed reduction and fixation with Kirschner wires or elastic stable intramedullary nails (ESIN). The inclusion criteria were: (1) skeletal immature, as determined by the presence of open physes on the radiographs.; (2) complete MDJ fracture of the distal humerus; (3) successful closed reduction and fixed with either Kirschner wires or ESIN; (4) less than three days between injury and surgery; (5) time of follow up at least 12 months. The exclusion criteria were: (1) communited fractures or multi-fractures of the same limb; (2) pathological fractures; (3) diagnosed osteopathy or relevant systematic diseases; (4) another orthopaedic event involving the same limb before the end of follow-up (e.g., a second fracture); (5) incomplete medical record or insufficient time of follow-up.

All of the patients had AP and lateral radiographs at the time of admission. For patients that could not make standard radiographs due to local pain, standard radiographs would be taken under general anesthesia with intra-operative fluoroscopy before manipulation. MDJ fractures were classified as lateral oblique, medial oblique, and transverse. Line a, b and d were drawn as described above. A line parallel to line d was drawn at the higher intersection point of the fracture line and the margin of the humeral shaft, which was defined as c′. The ratio of c′/d (the length of c′ divided by the length of d) was calculated as described in c/d (Figure 1B).

**Figure 2:**
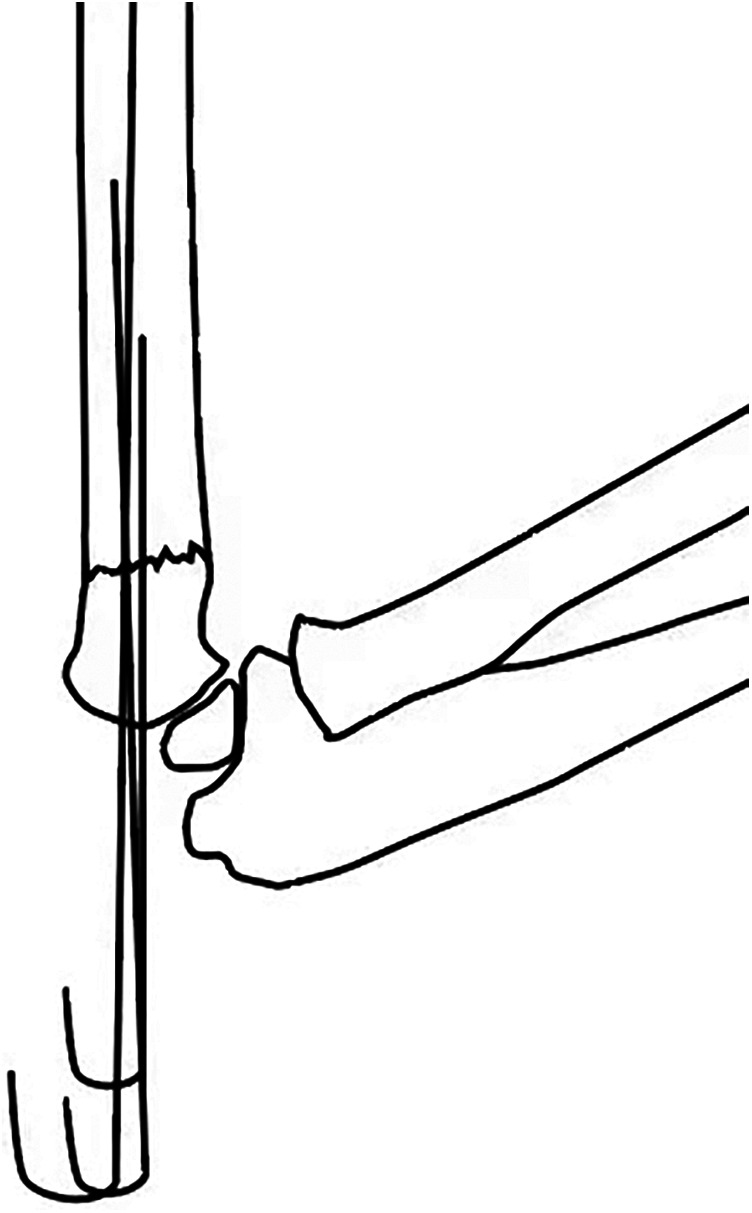
Illustration of the lateral view. By inserting the lateral pin lateral and posterior to the ossification center of the capitellum, a relatively “high” exit can be achieved.

**Figure 3:**
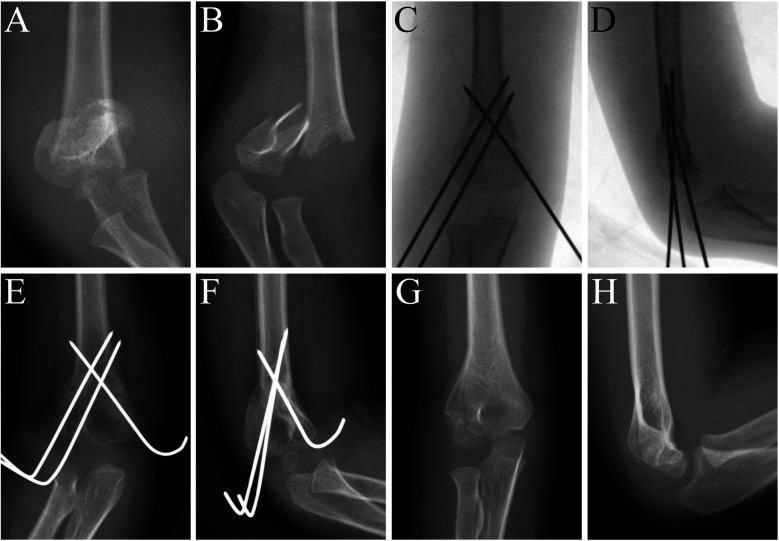
MDJ fracture in the right elbow of a 4-year-old boy. His c′/d was 1.51. He was fixed with crossed pinning. **(A)** AP view of the elbow before surgery. **(B)** Lateral view of the elbow before surgery. **(C)** AP view of the elbow immediately after surgery. **(D)** Lateral view of the elbow immediately after surgery. **(E)** AP view of the elbow 6 weeks after surgery. **(F)** Lateral view of the elbow 6 weeks after surgery. **(G)** AP view of elbow at 12 month follow-up after surgery. (**H)** Lateral view of elbow at 12 month follow-up after surgery.

**Figure 4:**
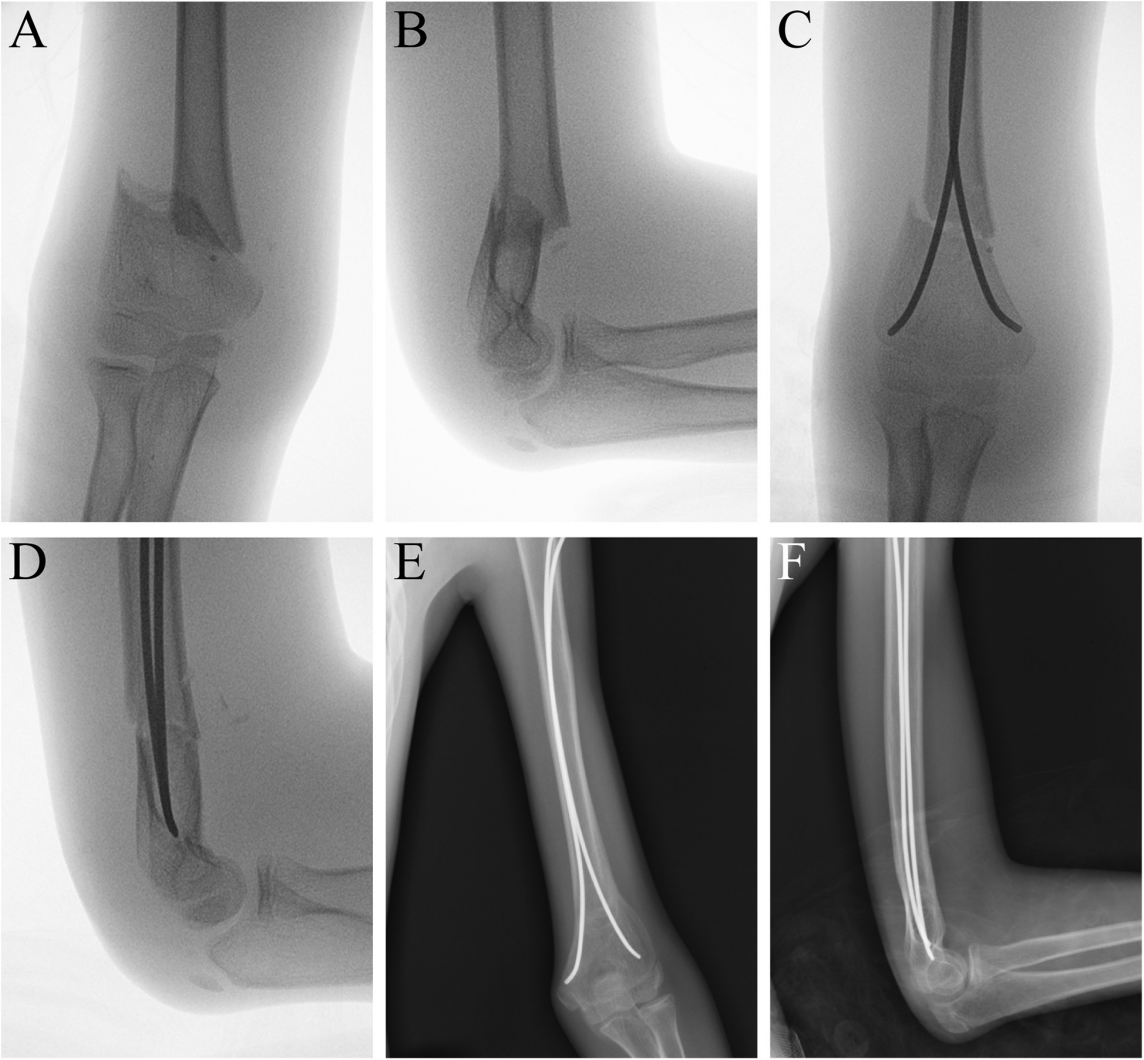
MDJ fracture in the left elbow of a 11-year-old boy. His c′/d was 1.19. He was fixed with two elastic stable intramedullary nails. **(A)** AP view of the elbow before surgery. **(B)** Lateral view of the elbow before surgery. **(C)** AP view of the humerus after surgery. **(D)** Lateral view of the humerus after surgery. **(E)** AP view of the humerus at 12 month after surgery. **(F)** Lateral view of the humerus at 12 month after surgery.

Based on the 140 normal radiographs included, the average c/d ratio was 1.31 ± 0.06, without much variation among different ages. However, in clinical practice, by inserting the lateral pin lateral and posterior to the ossification center of the capitellum, a more proximal pin exit above the upper border of the MDJ region could easily be achieved (Figure 2) (8). This exit can be much higher than that was designed in the bio-mechanical study and allows for more chance of fixing high MDJ fractures with pins. Since pinning has been advocated for economic concern, easy removal and the most important of all, the superiority in torsion control, we extended the usage of pins to c′/d ≧ 1.2 (9). For c′/d ≥ 1.2, Kirschner wires were used (Figure 3) and for those <1.2 ESINs were used (Figure 4) (8). For the Kirschner wire (KW) group, the arm was immobilized in an above-elbow plaster cast for 4–5 weeks postoperatively. For the ESIN group, the immobilization time was 3 weeks with a sling until bony callus was visible. When bone union was confirmed, KW would be removed under local anesthesia. ESINs would be removed under general anesthesia 6–12 months after the surgery. Non-weight-bearing activities of the elbow were encouraged right after the removal of cast. Full activities would be achieved gradually.

The patients were followed-up for at least 12 months after surgery. Detailed information on the demographic information of the patients, the fracture pattern and post-operative events was recorded. The functional outcome of the elbow was assessed according to the Flynn criteria. The carrying angle and range of motion were measured. Radiographs were evaluated for fracture healing, loss of fixation (defined as ≥5 mm of pin migration or ≥5 degrees of angular displacement in any direction on either the AP or lateral follow-up radiograph) (8), Baumann's angle and the lateral humeral-capitellar angle.

The Statistical package for social science (SPSS) 22.0 (SPSS Inc., Chicago, Illinois) was used for statistical analysis. Data were presented as mean ± SD (range). Chi-square test was used to compare the results between groups. *P* < 0.05 was considered significantly different.

## Results

**Figure 5:**
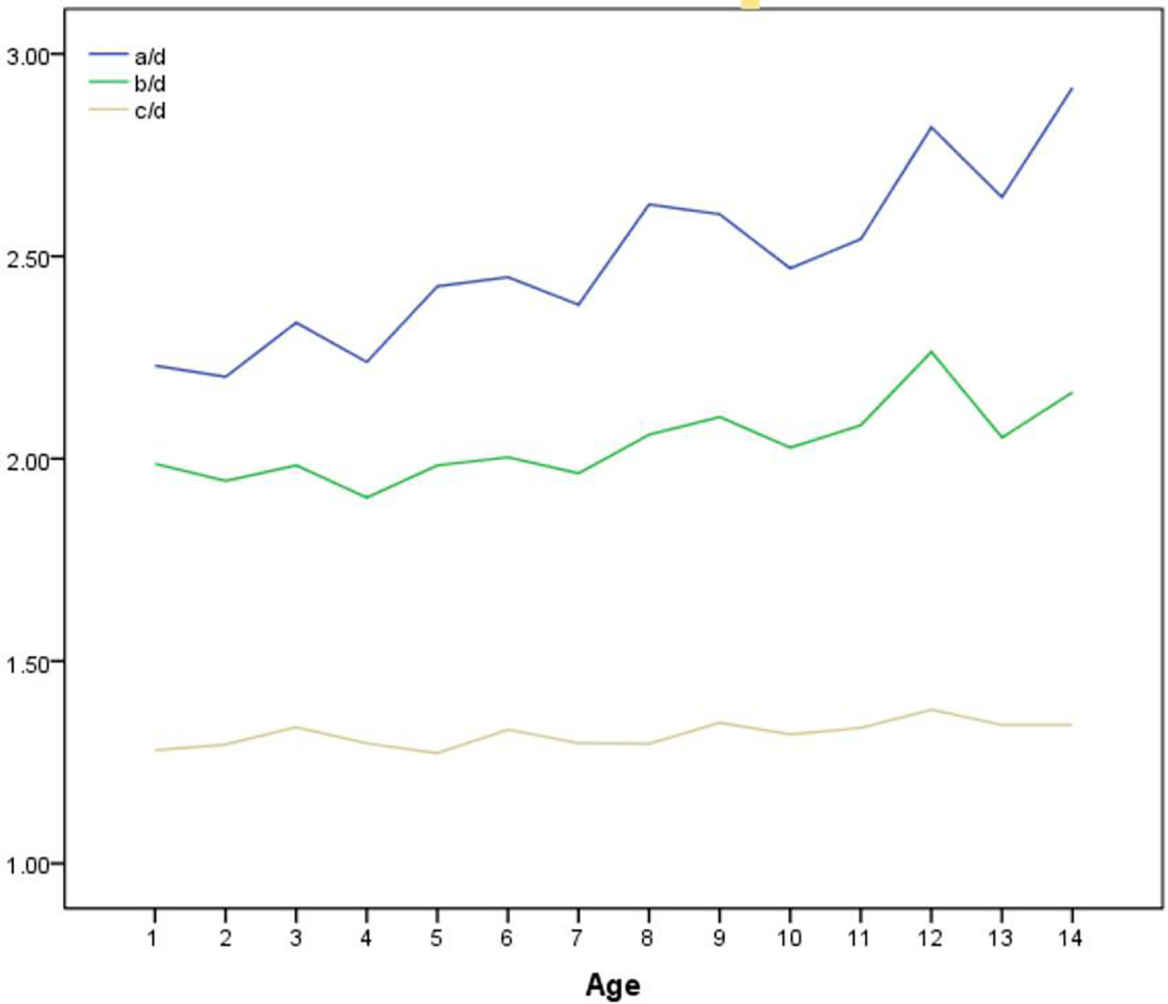
The average a/d, b/d and c/d measured on 144 normal elbow x-rays.

Altogether 140 normal standard radiographs were analyzed, with ten radiographs of different individuals in each age group and the gender ratio 1:1. The variation of a/d, b/d and c/d with age was shown in Figure 5. The mean a/d ratio was 2.49 ± 0.29, with slight increase as age grows. The average b/d and c/d ratio were 2.04 ± 0.18 and 1.31 ± 0.06 respectively, without much variation among different ages.

89 cases were finally included. Demographic information concerning the patients and the surgeries are listed in Table 1. Generally, there was no significant difference in outcomes between the two groups (*p* > 0.05). Although pin tract infection took place in the KW (5 cases) groups, they all healed uneventfully after local sterilization.

**Table 1:** Demographic and clinical parameters of children with MDJ fractures.

Parameters	KW (*n* = 73)	ESIN (*n* = 16)
Age, years	5.9 ± 2.7 (1–12)	6.3 ± 3.0 (3–12)
Sex, male/female	50/23	14/2
Fracture side, L/R	36/37	9/7
Duration of surgery (min)	59.5 ± 28.9 (10–168)	115.6 ± 43.2 (64–231)
Hardware removal	5.5 ± 0.7 (4–6) weeks	11.1 ± 2.0 (8–14) months
Pin tract infection	5	0
Follow up time (months)	14 ± 5.0 (12–39)	21 ± 14.5 (12–52)
Fracture line pattern	lateral oblique (22/73, 30.1%), medial oblique (4/73, 5.5%), transverse (47/73,64.4%)	lateral oblique (6/16, 37.5%), medial oblique (2/16, 12.5%), transverse(8/16, 50%)
Pre-operative Nerve injury	11 Radial nerve 7 Ulnar nerve 2 Medial nerve 2	0
c′/d	1.4 ± 0.6	1.1 ± 0.1
Baumann angle on the immediate postoperative radiograph (°)	75.7 ± 3.0	75.6 ± 3.8
Baumann angle at the last follow-up (°)	75.1 ± 4.8	76.4 ± 4.2
Humerocapitellar angle on the immediate postoperative radiograph (°)	42.3 ± 7.2	38.5 ± 7.1
Humerocapitellar angle at the last follow-up (°)	38.8 ± 5.4	38.6 ± 7.0
Carrying angle (°)	11.1 ± 4.0	10.3 ± 5.1
Contralateral Carrying angle (°)	12.6 ± 2.8	12.9 ± 2.8

Among the 73 (73/89.82%) cases fixed with Kirschner wires, 47 (47/73.64%) were transverse fractures. They were fixed with lateral or crossed pinning according to the experience and preference of the surgeon. For medial oblique fractures, crossed pinning was preferred due to the difficulty in achieving ideal distribution at the fracture site by lateral pinning only. For lateral oblique fractures, lateral pinning and crossed pinning were used to achieve sufficient stability (Table 2).

**Table 2:** Configurations for MDJ fractures fixed with Kirschner wires.

Fracture patterns	Number of cases (% of total)	Two lateral pins (2l)	Three lateral pins (3l)	Three crossed pins (3C) (Two lateral pins and one medial pin)	Four crossed pins (4C) (Two lateral pins and two medial pins)
Transverse fracture	47 (64.4%)	4 Excellent (4/4, 100%)	19 Excellent (17/19, 89.5%) Good (2/19, 10.5%)	19 Excellent (15/19, 78.9%) Good (2/19, 10.5%) Fair (2/19, 10.5%)	5 Excellent (3/5, 60%) Good (2/5, 40%)
Medial oblique fracture	4 (5.5%)	0	0	4 Excellent (4/4, 100%),	0
Lateral oblique fracture	22 (30.1%)	2 Excellent (2/2, 100%)	18 Excellent (16/18, 88.9%), Good (2/18, 11.1%)	1 Excellent (1/1, 100%)	1 Good (1/1, 100%)

According to Flynn, most of the patients had excellent elbow function at the last follow-up. Nine (12.3%) patients in the KW group and two (12.5%) patients in the ESIN group had loss of the carrying angle 6–10° and were ranked as good. Two (2.7%) patients in the KW group and one (6.3%) patient in the ESIN group had a loss of carrying angle 11–15° and was ranked as fair. There was no significant difference between the two groups (*p* = 0.423) (Table 3).

**Table 3:** Ranks of the elbows according to the Flynn criteria in the last follow-up.

Rank	KW (*n* = 73)	ESIN (*n* = 16)
Excellent	84.9% (62)	81.3% (13)
Good	12.3% (9)	12.5% (2)
Fair	2.7% (2)	6.3% (1)
Poor	0	0

## Discussion

MDJ fracture of the humerus is always posing difficulties to surgeons, in that the high fracture line is hard for Kirschner wires to reach more proximally while the distal part is two short for the ESIN to get enough control (10). It always takes the surgeons extra time to test for an ideal fixation strategy and therefore the operation time and concomitant injury may increase. In this case, a proper initial choice of fixation strategy would benefit the patients and the surgeons as well. Our previous study has provided theoretical basis for the selection, and this study moved on to test the recommendations in MDJ patients in clinical practice.

To date, this is the largest cohort of pediatric MDJ fractures. MDJ fractures in this series are all totally displaced, which approximated account for 8.24% of all Gartland type 3 supracondylar fractures (99/1201), which take up a higher percentage than 3% as reported by Fayssoux but a lower percentage than 12.4% as reproted by Park (2, 11). Male dominance is more obvious compared to that previously reported in all supracondylar fractures (M:F = 64:25), but both sides are similarly involved (L:R = 45:44) (12). Nerve injury was seen in 12% of all MDJ fractures (11/89), which is close to the 16% that was reported in humeral supracondylar fractures that require surgical treatment (13). In MDJ fractures the radial nerve is the most frequently injured (7/89), compared to the median nerve in common supracondylar fractures (14). Age seems to have no correlation with the fracture location of MDJ fractures. Ten cases (10.1%, 10/99) are comminuted and not included in the final statistical analysis.

**Figure 6:**
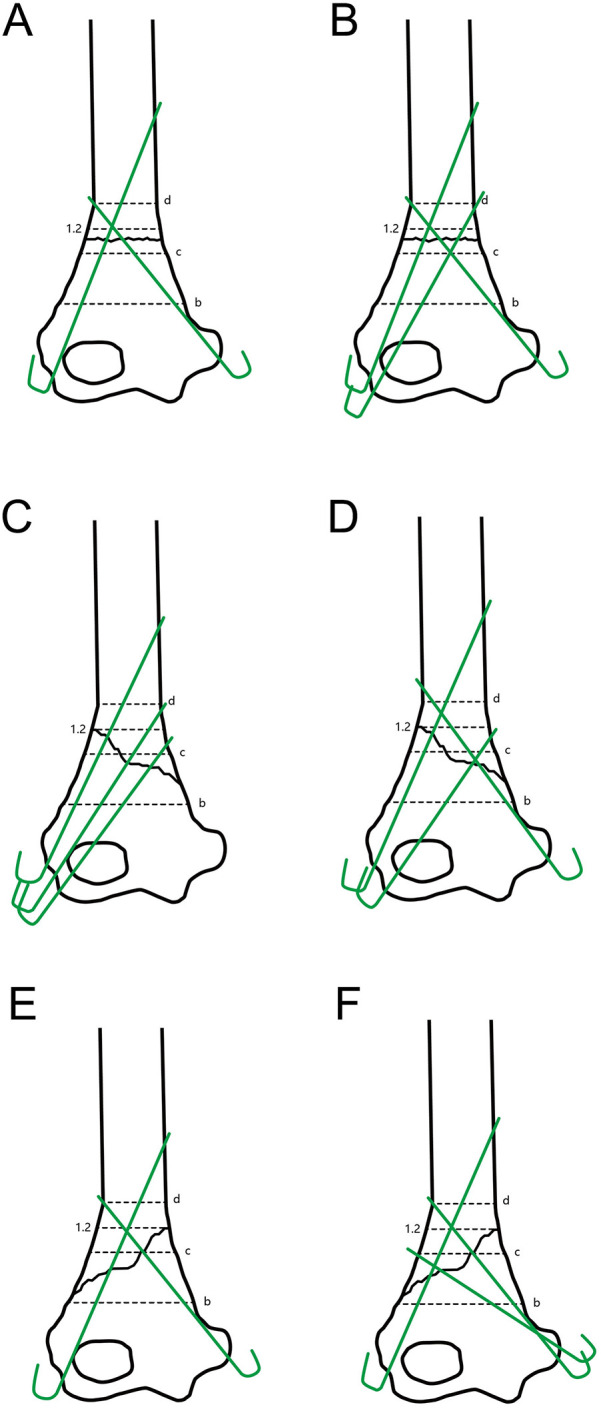
Illustration of the frontal view. **(A,B)** Pin distributions suggested for the transverse type; **(C,D)** pin distributions suggested for the lateral oblique type; **(E,F)** pin distributions suggested for the internal oblique type of MDJ fractures.

All but two cases fixed with pins had excellent or good functional outcome and experienced the shortest operation time. Superficial pin track infection took place in five cases, and they both healed with local sterilization after pin removal. When the fracture line was transverse, lateral or combined (lateral and medial) entry would be used according to the preference of the surgeon. When the fracture line tilted towards the ulnar side, namely the lateral oblique fracture (1), three divergent pins entering laterally would be chosen because this strategy can yield the best pin divergence at the fracture line. This could be easily achieved even if the c′/d was quite close to 1.2. For the medial oblique type, in which the fracture line tilted towards the radial side, such fixation might not be feasible, for the pin that went through the lateral column would form such a small angle with the fracture line that it could only fix a tiny piece of the proximal part (15). In this case one medial pin would be used in combination with two lateral pins. In several cases, we used two medial pins together with one or two lateral pins in order to achieve the maximus separation of the pins at the fracture line (Figure 6) (9). Although the medial condyle has limited space and bears the risk of ulnar nerve injury, two pins can be safely inserted under direct vision by making a small incision at the medial epicondyle. None of our cases treated with medial pinning had any sign of ulnar nerve injury after operation. One transverse fracture had an insufficient restoration of the Baumann angle during operation. Therefore, although this case had no post-operative displacement, it was ranked as fair in the follow-up.

For higher fracture lines, the pins have to form an acute angle with the humeral shaft to achieve stability, which carries a risk of slippage on the opposite inner cortex that may jeopardize stability of the construct (16). According to our previous findings, ESIN was used for cases with c′/d < 1.31 (7). ESIN has long been suggested in the treatment of supracondylar humeral fractures, especially those with difficulty in reduction and fixation. It was advocated for quick return to daily activity with no need for plaster/splint fixation (17). Its usage in MDJ fractures had also been suggested (18, 19). In such cases, the fracture line was more proximal, allowing for enough lever arms for ESIN to stabilize the distal part of the fracture. In biomechanical studies, ESIN showed the best stability against sagittal and coronal forces and comparable stability against torsional force among the common fixations (7). In clinical settings, ESIN also yielded excellent and good results in 15 out of our 16 patients. However, according to our experience, notice should be taken to ensure that the distal end of the medial nail is safely inserted into the metaphyseal bottom to firmly support the medial column. Otherwise, the frictional force would be insufficient to prevent redisplacement and medial column shortening, probably leading to cubitus varus.

There are always some cases in which the fracture is comminuted and hard to define the exact location. In those cases, both pin and ESIN fixation might experience some difficulty due to the unstable nature of the fracture site. In these cases, the lateral external fixator may be applied, with an additional radial or ulnar Kirschner wire depending on the preference of the surgeon and the track of the fracture line (5, 20). Meanwhile, external fixator may work well for all MDJ fractures, although it does not show any superiority over other fixations in biomechanical tests (7). As to the extra Kirschner wire, the ulnar entry has been reported to provide more torsional reliability than the radial entry with increased risk of ulnar nerve injury (21, 22). Besides, external fixators may raise the concerns of superficial/deep infection and inconvenience in daily life. However, in our cases (data not included), none of the patients treated with an EF had any sign of infection, redisplacement or nerve injury.

This study has some limitations. Although it is designed on the basis of our previous biomechanical research, the patients were not treated strictly according to the results of previous research. Different surgeons had their specific preference in pinning strategy that may influence the homogeneity of treatment. Besides, the sample sizes of one single center are relatively limited and unevenly distributed among groups, so that valid statistical comparisons cannot be made.

## Conclusion

Based on the largest cohort of humeral MDJ fractures reported in literature, our study showed a satisfactory short-term outcome according to our biomechanical- based management principles. ESINs were used for higher fractures, defined as c′/d < 1.2. Three lateral divergent or crossed pins were used for lower fractures with c′/d ≧ 1.2. In this way, most of the patients would achieve excellent to good outcome.

## Data Availability

The raw data supporting the conclusions of this article will be made available by the authors, without undue reservation.
